# The Hidden Berry, a Vascular Mystery: Unveiling a Comparison Between Two Cases of Orbital Cavernous Venous Malformation (OCVM)

**DOI:** 10.7759/cureus.85765

**Published:** 2025-06-11

**Authors:** Xin Yi Kong, Ummi Faradiana Abdul Rahim, Wan Mariny W Md Kasim

**Affiliations:** 1 Ophthalmology, Hospital Raja Permaisuri Bainun, Ipoh, MYS; 2 Ophthalmology, Hospital Serdang, Kajang, MYS

**Keywords:** case series, histopathology, orbital cavernous venous malformation, proptosis, surgery

## Abstract

Orbital cavernous venous malformations (OCVMs) are rare, benign vascular lesions found within the orbit, characterized by a cluster of dilated venous spaces surrounded by a fibrous capsule. These malformations can pose significant challenges in diagnosis and management due to their varied clinical presentations, ranging from subtle proptosis and visual disturbances to more severe complications such as orbital hemorrhage and nerve compression. The etiology of OCVMs remains largely unknown, though they are believed to be congenital in nature.

Case 1 was a 31-year-old male patient who had experienced painless swelling and blurred vision in his left eye for 11 years, which gradually worsened after a stone struck his left eye. He presented with a painful, red left eye for five days. On presentation, there was a significant left eye exophthalmos with lagophthalmos. He was also treated for exogenous endophthalmitis due to a corneal ulcer. Computed tomography (CT) demonstrated a large multilobulated intraorbital soft tissue mass. Tumor debulking via a transconjunctival approach was done. Histopathology confirmed orbital cavernous venous malformation. His left eye vision remained poor due to the past infection and optic nerve compression. One month post operation, it was noted that his left eye had developed recurrent ocular cavernous venous malformation. Case 2 was a 42-year-old female patient who presented with painless swelling for three months, with insidious onset and double vision. There were no thyroid or constitutional symptoms. She had diplopia on left lateral gaze, levoelevation, and levoversion, with restriction of the extraocular muscles in the superotemporal, superonasal, and nasal directions. On examination, axial proptosis was noted, but there was no lagophthalmos. Anterior and posterior chamber findings were unremarkable. CT showed a well-defined extraconal soft tissue lesion at the medial aspect measuring 2.1cm x 1.4cm x 1.6cm. The patient underwent excisional biopsy via anterior orbitotomy through vertical lid split. Histopathology reported as orbital cavernous venous malformation. The patient is recovering well.

The vital and functional prognosis with complete excision of OCVMs is generally excellent, but the complications from untreated proptosis can be devastating.

## Introduction

Vascular anomalies encompass a broad range of clinical presentations resulting from abnormalities in the blood or lymphatic vessels. These conditions are primarily categorized into tumors, such as hemangiomas, which exhibit high mitotic activity and endothelial proliferation, and malformations, which have normal mitotic rates and lack hypercellularity or accelerated cell turnover [1]. Orbital cavernous venous malformations (OCVMs), once referred to as cavernous hemangiomas, remain the most prevalent benign, non-infiltrative, slowly progressive vascular neoplasm found within the orbit. They are characterized by a cluster of dilated venous spaces surrounded by a fibrous capsule. OCVMs account for approximately 5% to 8% of all orbital tumors, with the majority of cases occurring in individuals between the ages of 30 and 50 years, and a higher prevalence is observed in women compared to men due to the expression of progesterone receptors in the epithelial cells of OCVMs [2,3]. These malformations can pose significant challenges in diagnosis and management due to their varied clinical presentations, ranging from subtle proptosis and visual disturbances to more severe complications such as orbital hemorrhage and nerve compression. The etiology of OCVMs remains largely unknown, though they are believed to be congenital in nature.

Histopathological examination of the tumor reveals a delicate capsule enclosing a mass composed of large, endothelial-lined vessels, with significant smooth muscle present in the vascular walls and surrounding stroma. OCVMs expand through the gradual enlargement of these thin-walled vascular channels, leading to the development of clinical symptoms [4]. Early diagnosis is essential to avoid unnecessary treatments and ensure appropriate management strategies. OCVMs can be found throughout the orbit, though more than 80% are typically situated within the intraconal compartment, particularly in the lateral region [5]. On CT imaging, these hemangiomas appear as well-circumscribed, homogeneous masses with a slightly hyperdense texture relative to the adjacent ophthalmic muscles. On magnetic resonance imaging (MRI), cavernous hemangiomas exhibit a heterogeneous signal on both T1- and T2-weighted images, with mild enhancement observed on T1-weighted images following contrast administration [6]. OCVMs consistently appear as well-circumscribed, ovoid masses with flow voids within the intraconal space, characterized by a markedly homogeneous hyperintense signal on T2-weighted imaging [7].

The most recent classification system, developed by the International Society for the Study of Vascular Anomalies (ISSVA), serves as the foundation for diagnosing and managing vascular lesions, including those occurring in the orbit. Treatment strategies for OCVMs vary widely, depending on the size, location, and symptoms of the lesion. While asymptomatic lesions are often managed conservatively, symptomatic cases may require surgical intervention, sclerotherapy, or embolization. The risk of complications, including hemorrhage and nerve damage, necessitates careful consideration in management.

Our case series highlights the diverse clinical spectrum of OCVMs through two distinct presentations with varying severity, complications, and surgical outcomes. The first case involved a long-standing, 11-year history of painless swelling that acutely worsened after trauma, leading to no perception of light (NPL) with relative afferent pupillary defect (RAPD) positivity due to optic nerve compression. This case was further complicated by white cataract, exposure keratopathy, and exogenous endophthalmitis, requiring additional medical management. In contrast, the second case presented with an insidious onset over three months, maintaining good visual acuity with no RAPD but experiencing diplopia, which fully resolved postoperatively. The tumors also differed in location and surgical complexity. Case 1 had a large intraorbital OCVM extending to the orbital apex, making complete resection unfeasible and leading to tumor recurrence, whereas Case 2 had a well-encapsulated extraconal lesion in the medial orbit, allowing for complete excision with no recurrence at 10 months. These cases underscore the variability in OCVM presentations, the role of complications in disease progression, and the importance of early detection, individualized surgical planning, and long-term follow-up to optimize patient outcomes. By sharing our experience, we aim to expand the existing knowledge base and offer valuable guidance for clinicians encountering similar atypical presentations.

## Case presentation

This is a case series of two patients in the age range of 30-45 years and of different genders. Both patients originally came as outpatients to the ophthalmology clinic due to slow-progressing proptosis. This case series was conducted following ethical guidelines in accordance with the principles of the Declaration of Helsinki, with patient confidentiality and informed consent being upheld. Informed consent was obtained from both patients for the publication of their clinical information and images in this case series.

Case 1

**Figure 1:**
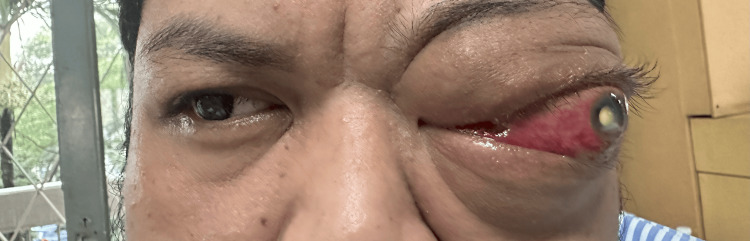
The patient with left eye non-axial proptosis with white cataract and exposure keratopathy.

**Figure 2:**
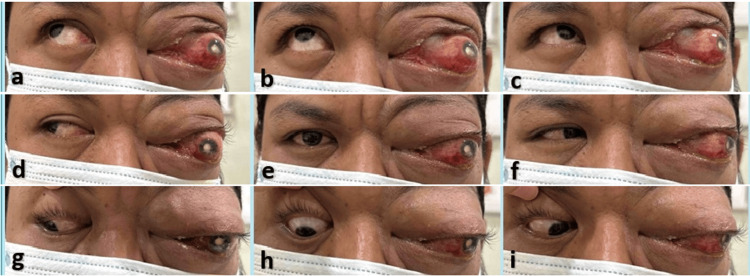
A nine-gaze picture of the patient shows restriction of the extraocular muscles in all gazes of the left eye. A. dextroelevation; B. elevation; c. Levoelevation; D. dextroversion; E. primary position; F. levoversion; G. dextrodepression; H. depression; I. levodepression

**Figure 3:**
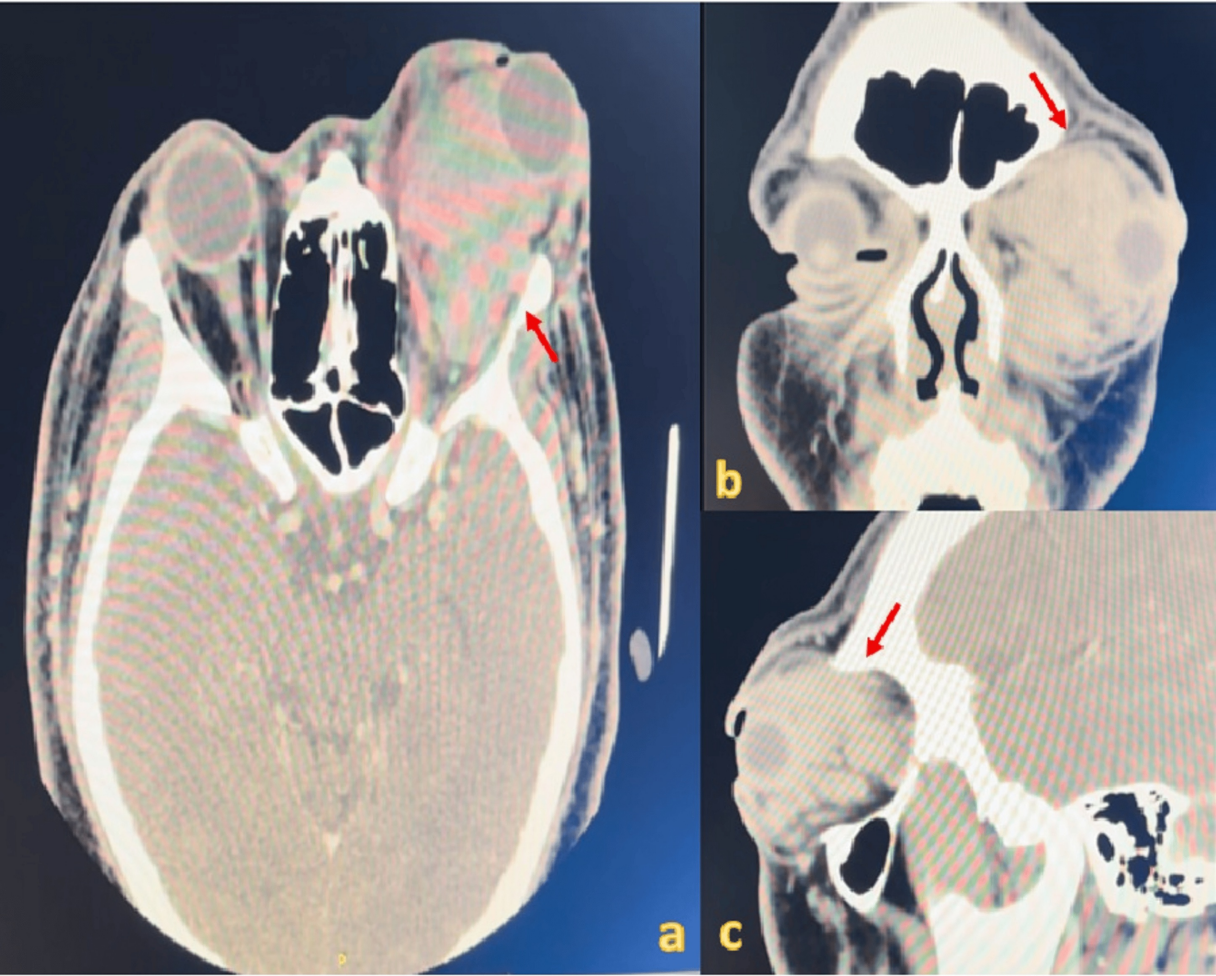
Computed tomography (CT) of the brain and orbit a. CT in axial cut; b. CT in coronal cut; c. CT in sagittal cut. The red arrow shows a large, multilobulated intraorbital soft tissue mass.

**Figure 4:**
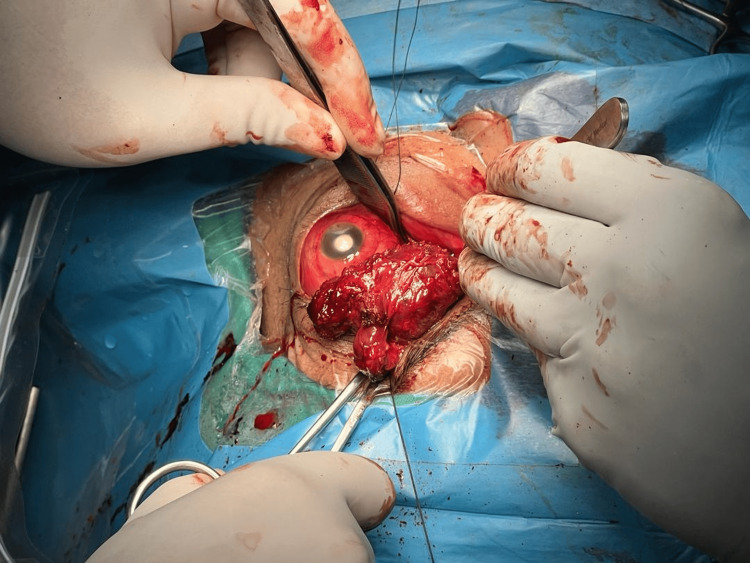
Left eye tumor debulking via transconjunctival approach. The patient was made to lie flat. Medial limbal conjunctival peritomy was done 180 degrees with a relaxing incision tangentially. Blunt dissection was done until the mass was identified and the adherent tissue was released. The medial rectus was then identified, and the mass was clamped and excised. A drain tube was inserted.

**Figure 5:**
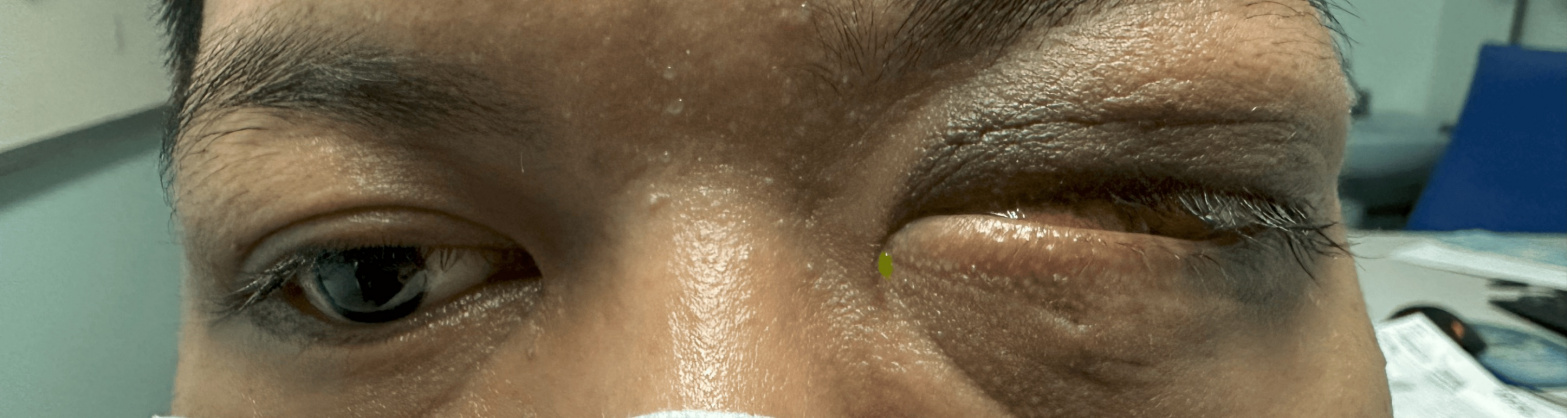
One month postoperative photograph showed reduced swelling, but the superior mass was noted to be slightly increased. The computed tomography (CT) scan revealed recurrence.

A 31-year-old male patient presented with painless swelling and blurring of vision in his left eye for 11 years, which gradually worsened after a stone hit his left eye. He experienced a painful, red eye for five days. On presentation, there was a significant left eye exophthalmos with lagophthalmos. In addition, he also had a white cataract over his left eye with exposure keratopathy. His visual acuity was NPL with RAPD positive over the left eye. The extraocular muscle movement for the left eye was restricted at all gazes (Figures 1, 2). He was also treated for exogenous endophthalmitis due to a corneal ulcer. Computed tomography (CT) demonstrated a large multilobulated intraorbital soft tissue mass (Figure 3). Tumor debulking via a transconjunctival approach was performed (Figure 4). Intraoperative findings showed multiple irregular, firm, and reddish masses. There were two masses with the size of 4cm x 5cm x 2cm and one mass measuring 2cm x 1.5cm x 1cm. Ninety percent of the tumor was removed; however, 10% of it was located near the apex and was attached to the optic nerve, hence unable to be removed. The patient was discharged well from the hospital five days after the operation, when there was no more in the drain tube, and the tube was removed. He was discharged with ointment Maxitriol and artificial tears for his left eye. Since there was only minimal postoperative lagophthalmos, there was no role for tarsorrhaphy. The patient came back two weeks after the operation for an appointment, and his exophthalmos was greatly reduced, and the exposure keratopathy was resolved. Histopathology confirmed it as an OCVM. His left eye vision remains the same, which is NPL due to the previous infection and optic nerve compression (Figure 5). Upon the latest follow-up, it was noted that his left eye developed recurrent OCVM, but the patient opted against further surgery and subsequently defaulted on his follow-up. 

Case 2

**Figure 6:**
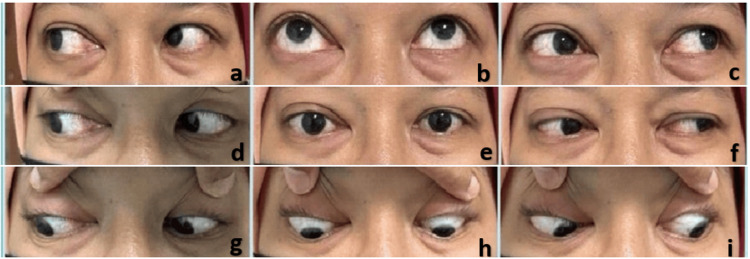
Nine-gaze picture of the patient showing minimal restriction of the right eye of dextroelevation, elevation, levoelevation, and levoversion. A. dextroelevation; B. elevation; c. levoelevation; D. dextroversion; E. primary position; F. levoversion; G. dextrodepression; H. depression; I. levodepression

**Figure 7:**
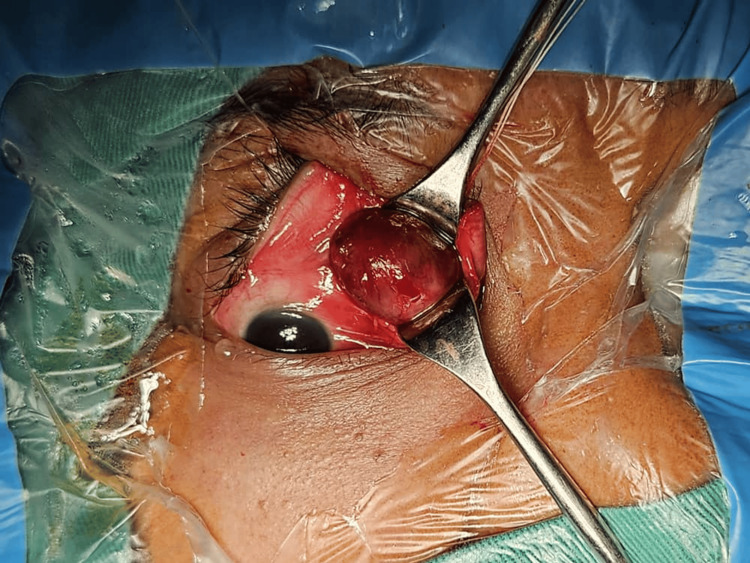
Anterior orbitotomy through a vertical lid split

**Figure 8:**
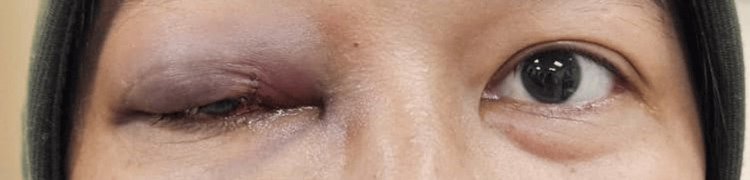
A photograph taken one week postoperatively showed slight swelling and erythema of the right eyelid.

**Figure 9:**
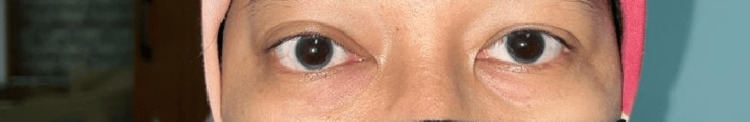
A photograph taken six weeks postoperatively showed no swelling or redness.

A 42-year-old female patient presented with painless swelling in her right eye for three months, with insidious onset and double vision. There were no thyroid or constitutional symptoms. Upon examination, her visual acuity was 6/9 with RAPD negative. Axial proptosis over the right eye was noted, but there was no lagophthalmos. She experienced diplopia on left lateral gaze, levoelevation, and levoversion, with restriction of extraocular muscle over superotemporal, superonasal, and nasal areas (Figure 6). Anterior and posterior chamber findings were unremarkable (Figure 7). CT showed a well-defined extraconal soft tissue lesion at the medial aspect, measuring 2.1cm x 1.4cm x 1.6cm. The patient underwent excision biopsy via anterior orbitotomy through vertical lid split. Intraoperative findings showed a well-encapsulated, solitary, soft, and firm reddish mass with the size of 2cm x 2 cm, and it was excised in full. The patient was discharged two days after the operation with Maxitriol ointment three times per day. Two weeks post operation, the patient was seen back in the clinic, and histopathology confirmed it as an OCVM. The patient recovered well, with no signs of recurrence and resolved diplopia 10 months postoperatively (Figures 8, 9). 

## Discussion

OCVM is a benign vascular disease characterized by abnormal changes in the vascular cavity. It is a rare but most common benign vascular tumor, representing between 4.5% and 7.4% of all orbital tumors [8]. Approximately 60% of cases are observed in women, and recent studies indicate that female sex hormones may play a role in influencing the clinical progression of OCVM [9]. The lesion may also express vascular endothelial growth factor receptor 2 and progesterone receptors. These observations support the hypothesis that neoangiogenesis, triggered by intraluminal thrombosis, contributes to the gradual progression of OCVM, which could explain the reported progression during pregnancy [10]. Therefore, there is a risk factor for developing OCVM in our second case. It is a slow-growing tumor with a radiographic growth rate of about 10% to 15% per year [11], which has a lasting effect on visual function and ocular motility. OCVM primarily tends to occur in the middle third of the orbit, with a higher frequency in the intraconal space. Within intraconal lesions, some studies report a greater incidence of lesions located lateral to the optic nerve [12]. As OCVM often occurs in the muscle cone behind the eyeball, it can cause axial exophthalmos and gradually compress the optic nerve or eyeball, resulting in loss of vision. Visual deterioration following exophthalmos is the second most common sign of CVM. In larger cohorts, approximately 50% of patients experience varying degrees of visual impairment, typically no worse than 20/40. Motility deficits and manifest strabismus in the primary position are observed in about 20% to 30% of cases. Additionally, choroidal folds (retinal striae) and optic disc abnormalities, such as elevation or blurring of the disc margins, optic nerve head vein dilation, and optic atrophy due to mass effect, may be present. Mechanical ptosis and corneal exposure are uncommon [2]. This statement is slightly deviated from both cases mentioned above. That is why we are reporting these two cases to showcase the uncommon presentation of OCVM. The tendency for OCVM to predominantly affect the left orbit continues to elude explanation [13], as demonstrated in only one of our cases.

A CT scan typically reveals a well-circumscribed mass with homogeneous soft-tissue density and, in rare cases, possible bone remodeling or erosion. Contrast enhancement varies depending on the imaging phase, ranging from focal early enhancement to heterogeneous intermediate and late diffuse enhancement. MRI on T1-weighted images shows the lesion as isointense to muscle and gray matter and hypointense to fat. On T2-weighted images, it appears hyperintense compared to fat and brain tissue. With contrast enhancement, the pattern becomes progressively more uniform over time, transitioning from patchy and heterogeneous to a more consistent appearance, although larger lesions may retain a heterogeneous pattern in the later phases. Solitary fibrous tumors, lymphatic malformations, orbital lymphoma, and schwannomas can all resemble OCVM and may be difficult to differentiate from it [2]. As for histological and morphological features, OCVM is a benign vascular malformation consisting of large vascular channels lined with endothelial cells and abundant stroma. The vascular lumen contains blood and areas of intralesional thrombosis, indicating slow blood flow. Endothelial cells resemble mature vascular elements, while the stroma exhibits perivascular hypercellularity or neovascular activity [11]. A characteristic feature of OCVM is its well-defined, compact capsule, which differentiates orbital venous malformations from those in subcutaneous or hepatic tissues. [14] Therefore, orbital imaging, a combination of ultrasound, CT, and MRI, helps with precise diagnosis in most cases, but pathology ultimately confirms the diagnosis [2].

Surgical treatment for OCVM is typically necessary for symptomatic cases, optic nerve compression, or disfiguring proptosis, while observation is appropriate for asymptomatic cases due to the rare occurrence of acute bleeding and slow progression. The choice of approach for removal depends on the lesion's anatomical location and relationship with surrounding orbital structures, with the surgeon's experience being a key factor in deciding the appropriate neurosurgical, otolaryngological, or ophthalmological approach. Recently, anterior orbitotomy has become more commonly utilized for the removal of both extraconal and intraconal OCVM lesions that do not involve the orbital apex, accounting for nearly 60% of cases. This can be performed via either transconjunctival or transcutaneous methods, as these approaches offer comparable surgical outcomes with shorter operative times and less tissue dissection, whether transconjunctival or transcutaneous, which was done in both cases, as these methods offer comparable surgical outcomes with shorter operative times and less tissue dissection [15]. 

However, there are other treatment options. Sclerotherapy is a minimally invasive treatment for patients who are either unwilling to undergo surgery or have OCVMs located in deep orbital positions, where excision could damage surrounding structures such as extraocular muscles, nerves, and the optic nerve [16]. Intralesional injection of pingyangmycin (bleomycin A5) into low-flow orbital vascular lesions, such as OCVM, induces endothelial cell apoptosis, leading to sclerostenosis and a significant reduction in lesion volume, proptosis, swelling, and pain [17]. As for the recurrence of OCVM, it may be due to either a second, smaller preexisting lesion or an incomplete resection of the primary lesion. However, there are reports in the literature of "recurrent" CVM occurring even after what appeared to be a complete surgical excision [18], as happened in Case 1, but not in Case 2.

Traditional fractionated radiotherapy poses a significant risk to the optic nerve, as it receives nearly the same radiation dose as the target lesion. In contrast, stereotactic radiosurgery (SRS) administers a precise, high-dose radiation treatment in a single session, minimizing exposure to surrounding structures and reducing collateral damage. Gamma knife radiosurgery (GKRS), a form of SRS, has shown to be beneficial in 96% of patients with orbital apex lesions, leading to significant improvements in vision, visual field, retinal nerve fiber layer (RNFL) thickness, proptosis, and diplopia, along with a substantial reduction in tumor volume after multiple sessions. Additionally, anecdotal small case series have reported tumor shrinkage in patients with orbital apex lesions treated with fractionated stereotactic radiotherapy, with no significant side effects. Following a course of fractionated stereotactic radiation, studies have documented an average tumor reduction of 60%, effectively preventing total blindness or further vision deterioration while improving the visual field. Fractionated radiation serves as an alternative treatment for inaccessible and unresectable tumors. While the precise mechanism of GKRS remains unclear, one hypothesis suggests that radiation induces thrombosis and subsequent fibrosis within the lesion, leading to closure of vascular spaces and tumor contraction. Additionally, due to venous stasis, cavernous malformations may be more susceptible to thrombosis, and radiation therapy may accelerate this thrombotic and fibrotic process, further contributing to lesion regression [19].

Furthermore, Putterman and Goldberg first described the use of cryoextraction for orbital malignancies in 1975 [20]. In a study by Castelnuovo et al., two cases of intraorbital intraconal hemangiomas were successfully excised using a transnasal approach, demonstrating excellent safety outcomes with the cryoprobe. Given that the hemangiomas were closely situated near blood vessels and nerves, the Joule-Thompson effect facilitated a temporary yet effective bond between the probe tip and the lesion’s surface. This allowed the surgeon to apply gentle traction, aiding in the separation of adhesions from surrounding tissues. Due to their effectiveness in removing fluid-filled intraorbital lesions, cryoprobes have become a valuable adjunct in the surgical management of orbital tumors [21].

## Conclusions

This case series highlights the diverse clinical presentations and outcomes associated with OCVMs. The contrasting scenarios, a long-standing lesion complicated by trauma-induced optic nerve compression versus an insidious, less complicated extraconal lesion, emphasize the significance of early detection and accurate characterization through imaging modalities. Individualized management plans, tailored to lesion location, symptom severity, and surgical accessibility, are crucial for optimizing visual and functional outcomes. Given the potential for recurrence and serious complications, including optic nerve compression and severe visual impairment, long-term patient follow-up is essential. Further studies involving larger cohorts and standardized protocols are recommended to refine management strategies and improve patient care in OCVM.

## References

[1] Mulliken JB, Glowacki J (1982). Hemangiomas and vascular malformations in infants and children: a classification based on endothelial characteristics. Plast Reconstr Surg.

[2] Calandriello L., Grimaldi G., Petrone G. (2017). Cavernous venous malformation (cavernous hemangioma) of the orbit: current concepts and a review of the literature. Surv Ophthalmol.

[3] Di Tommaso L, Scarpellini F, Salvi F, Ragazzini T, Foschini MP (2000). Progesterone receptor expression in orbital cavernous hemangiomas. Virchows Arch.

[4] Rosenblum B, Rothman AS, Lanzieri C, Song S (1986). A cavernous sinus cavernous hemangioma. Case report. J Neurosurg.

[5] Wilms G (2009). Orbital cavernous hemangiomas. AJNR Am J Neuroradiol.

[6] Vogele D, Sollmann N, Beck A (2022). Orbital tumors-clinical, radiologic and histopathologic correlation. Diagnostics (Basel).

[7] Xian J, Zhang Z, Wang Z (2010). Evaluation of MR imaging findings differentiating cavernous haemangiomas from schwannomas in the orbit. Eur Radiol.

[8] Ayoub E, Farid A, Yahya C (2022). Cavernous hemangioma of the orbit: case report and a review of the literature. Radiol Case Rep.

[9] Jayaram A, Lissner GS, Cohen LM, Karagianis AG (2015). Potential correlation between menopausal status and the clinical course of orbital cavernous hemangiomas. Ophthalmic Plast Reconstr Surg.

[10] Dinakar I, Naik RT, Purohit AK, Ratnakar KS (1993). Cavernous hemangioma of the orbit: a case report. Indian J Pathol Microbiol.

[11] Rootman DB, Heran MK, Rootman J, White VA, Luemsamran P, Yucel YH (2014). Cavernous venous malformations of the orbit (so-called cavernous haemangioma): a comprehensive evaluation of their clinical, imaging and histologic nature. Br J Ophthalmol.

[12] McNab AA, Wright JE (1989). Cavernous haemangiomas of the orbit. Aust N Z J Ophthalmol.

[13] McNab AA, Selva D, Hardy TG, O'Donnell B (2014). The anatomical location and laterality of orbital cavernous haemangiomas. Orbit.

[14] Rootman DB, Rootman J, White VA (2015). Comparative histology of orbital, hepatic and subcutaneous cavernous venous malformations. Br J Ophthalmol.

[15] Yang M, Yan J (2014). Long term surgical outcomes of orbital cavernous haemangiomas (low-flow venous malformations) as performed in a tertiary eye hospital in China. J Craniomaxillofac Surg.

[16] Rootman J, Heran MK, Graeb DA (2014). Vascular malformations of the orbit: classification and the role of imaging in diagnosis and treatment strategies*. Ophthalmic Plast Reconstr Surg.

[17] Chen Y, Li Y, Zhu Q, Zeng Q, Zhao J, He X, Mei Q (2008). Fluoroscopic intralesional injection with pingyangmycin lipiodol emulsion for the treatment of orbital venous malformations. AJR Am J Roentgenol.

[18] Yan J, Wu Z (2004). Cavernous hemangioma of the orbit: analysis of 214 cases. Orbit.

[19] Seher S, Khan SA, Mounien S (2023). Emerging trends & techniques related to treatment in patients with intra-conal cavernous hemangioma of the Orbit(OCH)/orbital cavernous malformation (OCVM) and treatment related complications, a systematic review. J Int Med Grad.

[20] Putterman A, Goldberg MF (1975). Retinal cryoprobe in orbital tumor management. Am J Opthalmol.

[21] Castelnuovo P, Arosio AD, Volpi L (2019). Endoscopic transnasal cryo-assisted removal of orbital cavernous hemangiomas: case report and technical hints. World Neurosurg.

